# Organic fertilization with cow dung modulates growth, bioactive compounds, and antioxidant capacity in duckweed (*Wolffia globosa*)

**DOI:** 10.3389/fpls.2026.1751657

**Published:** 2026-02-10

**Authors:** Nitesh Kumar Yadav, Arun Bhai Patel, Deepan Rajesh S., Pradyumn Kumar Parida, Sampa Baidya

**Affiliations:** College of Fisheries, Central Agricultural University (Imphal), Agartala, Tripura, India

**Keywords:** antioxidant activities, bioactive compounds, cow dung, duckweed, *Wolffia*

## Abstract

**Introduction:**

*Wolffia globosa* (wolffia) is a fast-growing aquatic plant rich in nutrients and bioactive compounds, whose biomass and functional quality are strongly influenced by fertilization. However, the effects of organic fertilizers on bioactive composition and antioxidant potential in wolffia remain poorly understood. This study fills this gap by evaluating fresh cow dung as an organic fertilizer and elucidating its novel role in enhancing biomass production, biochemical composition, bioactive compounds, and antioxidant capacity of wolffia.

**Methods:**

A randomized complete block design was employed with six treatments and three replications each. Treatments included an inorganic fertilizer control and five levels of cow dung applied at 10, 20, 30, 40, and 50 g L^–1^. Biomass yield was recorded, while biochemical composition (crude protein, crude lipid), bioactive compounds (total phenolics, flavonoids, carotenoids, tannins, ascorbic acid), and antioxidant activity (DPPH, ABTS, FRAP assays) were analyzed using standard protocols.

**Results:**

Biomass was highest at 30 and 20 g L^–1^ cow dung, reaching 152.38 ± 12.25 g and 144.88 ± 1.28 g, respectively. Crude protein content was also highest at 40 g L^–1^ (30.40%) and in the control (29.31%). Crude lipid content peaked at 40 g L^–1^ (5.09%) and 50 g L^–1^ (4.91%). The highest total phenolic content (350.04 mg GAE g^–1^), total flavonoid content (159.35 mg QE g^–1^), and ascorbic acid (99.05 mg 100 g^–1^) were recorded at 50 g L^–1^ cow dung. In contrast, total carotenoid content (2286.90 µg g^–1^) and chlorophyll-b (11.82 µg g^–1^) were significantly higher in the control. Total tannin content did not differ significantly among the control, 10, and 50 g L^–1^ treatments, whereas the 20 and 30 g L^–1^ treatments exhibited reduced levels. Antioxidant activity was highest at 50 g L^–1^ for both DPPH (57.42%) and ABTS (65.65%) assays, while FRAP activity peaked at 10 g L^–1^ (180.64 μmol Fe g^–1^).

**Discussion:**

The results demonstrate that cow dung enhances the bioactive compounds and antioxidant potential of wolffia. While medium doses (20–30 g L^–1^) maximized biomass production, higher cow dung levels, particularly 50 g L^–1^, promoted greater accumulation of phenolics, flavonoids, and overall antioxidant capacity.

## 1 Introduction

*Wolffia globosa* (wolffia), the smallest member of the duckweed family, is a free-floating, rootless aquatic angiosperm known for its rapid vegetative propagation with doubling times of just 2–3 days (Patel et al., 2022; Sree et al., 2015). Due to its exceptional nutritional profile, wolffia has garnered interest as a sustainable crop. It is rich in high-quality protein with a well-balanced essential amino acid composition, digestible starch, a high proportion of unsaturated fatty acids, and low levels of anti-nutritional factors (Yadav et al., 2024a). It has been utilized in various applications, including human consumption (Appenroth et al., 2018), and as feed for livestock and farmed animals such as ducks and fish, in both fresh and dried forms (Pradhan et al., 2019; Chantiratikul et al., 2010). Beyond its nutritional benefits, wolffia is also a rich source of bioactive compounds such as phenolics, flavonoids, alkaloids, carotenoids, and other antioxidants (Yadav et al., 2024b; Yadav et al., 2025a). These compounds contribute to its potential value in the food, feed, and pharmaceutical industries. Notably, the bioactive constituents of wolffia exhibit a range of pharmacological properties, including antibacterial, antidiabetic, antiviral, antifungal, antioxidant, anti-inflammatory, and anticancer activities (Yadav et al., 2025a).

The synthesis and accumulation of these bioactive compounds in wolffia are highly influenced by environmental and agronomic factors. Abiotic stresses such as changes in light intensity, temperature, salinity, and nutrient availability have been reported to enhance the production of both primary and secondary metabolites (Yadav et al., 2025b). Among these factors, nutrient supply plays a central role in plant growth, physiological activity, and biochemical composition.

Organic fertilizers, particularly animal manures such as cow dung, are rich sources of essential nutrients, including nitrogen (N), phosphorus (P), and potassium (K) (Nguyen et al., 2024). Key macronutrients such as N and K are critical determinants of crop yield, texture, nutritional quality, and post-harvest shelf life (Abdel-Gawad et al., 2025). Unlike inorganic fertilizers, organic manures not only support plant growth but also stimulate the biosynthesis of secondary metabolites (Rostaei et al., 2024). Numerous studies have demonstrated that the application of organic fertilizers can significantly enhance the levels of phenolic compounds, flavonoids, and antioxidants in various crops. For example, Pacheco et al. (2021) reported that the application of cattle and poultry manure increased polyphenol content by 46.83% and 69.64%, respectively, compared to the control, while cattle manure treatment further enhanced antioxidant activity in *Passiflora incarnata* L. leaves by 32.72% after 90 days of cultivation. increased by 32.72% following cattle manure treatment after 90 days of cultivation. Similarly, Poozesh and Amirahmadi (2024) observed a 203.23% increase in total phenolic content in *Zingiber officinale* Rosc. with poultry manure application compared to the control. Aina et al. (2019) also reported a notable enhancement in the accumulation of bioactive compounds in organically fertilized crops.

In light of increasing global concerns over the environmental and health impacts of synthetic agrochemicals, organic farming has emerged as a sustainable alternative that emphasizes ecological balance and reduced chemical inputs (Panday et al., 2024). The use of organic fertilizers aligns with this philosophy, offering a means to enhance not only plant productivity but also the nutritional and medicinal quality of harvested biomass (Parra-Pacheco et al., 2024). The concentration and composition of bioactive compounds serve as important quality indicators, particularly for nutraceutical and pharmaceutical applications (Pacheco et al., 2021).

Although the beneficial effects of organic manure on the accumulation of secondary metabolites have been demonstrated in various crops, limited information is available on its impact on wolffia. Therefore, the present study aimed to evaluate the effects of raw cow dung on multiple parameters in wolffia, including biomass production, biochemical composition, and the content of key bioactive compounds such as total phenolic content (TPC), total flavonoid content (TFC), total tannin content (TTC), total carotenoid content (TCC), and ascorbic acid. Antioxidant activities were also assessed using DPPH, ABTS, and FRAP assays.

## 2 Materials and methods

### 2.1 Plant material and treatments

The study was conducted in April 2023 at the College of Fisheries, CAU, Tripura (23°45’ N; 91°15’ E). Eighteen thermocol fish boxes (58 × 39 × 30 cm; 0.226 m² surface area), lined with transparent plastic film, were used under natural sunlight in a transparent polyhouse. Each box was cleaned and filled with 30 L of groundwater (12 cm depth). Fresh cow dung was added at 0 (control; inorganic fertilizer), 10, 20, 30, 40, and 50 g L^–1^ and mixed thoroughly with a wooden stick. Wolffia fronds (100 g), sourced from the College of Fisheries, Lembucherra, were inoculated into each box and cultured for a period of 7 days. In the control treatment, inorganic fertilizers were applied as urea (48 mg L^–1^), single super phosphate (SSP; 70 mg L^–1^), muriate of potash (MOP; 25 mg L^–1^), and a vitamin–mineral mix (150 mg L^–1^). Growth was monitored throughout the experimental period. At the end of the experiment, biomass was harvested using a hand or scoop net, weighed, oven-dried, and subsequently pulverized for further analyses.

### 2.2 Biomass and growth analyses of wolffia

Fresh biomass yield was calculated on a fresh-weight basis by subtracting the initial inoculum fresh weight from the total fresh biomass harvested. The samples were subsequently oven-dried at 50 °C for 24 h using a hot air oven (Yona, Indian Instruments Manufacturing Co., India), pulverized into a fine powder, and stored for further analyses. The specific growth rate (SGR) and net biomass were determined using Equations 1, 2, respectively (Said et al., 2022a).

(1) $$SGR\left(\%\right)=\frac{\left(Ln\;Wt-Ln\;W0\right)}{t}\times 100$$

Where Wt is wolffia biomass at time t,

W_0_ is wolffia initial biomass, and t is the length of cultivation (days).

(2) Net biomass (g)=biomass harvested (g) − biomass stocked (g)

### 2.3 Proximate composition analysis

Proximate composition of wolffia was analyzed following AOAC (2005) methods. Protein content was determined via Kjeldahl digestion (Kel Plus Kes 12b E, Pelican Equipments, India) using H_2_SO_4_ and K_2_SO_4_:CuSO_4_ (9:1) catalyst at 410 °C for 1 h 45 min, followed by distillation (Kjeltec 8400, FOSS, Denmark). Crude lipid was measured using the Soxtec (ST 243, FOSS) systems.

### 2.4 Sample extraction

Dried samples (100 mg) were extracted with 100 mL distilled water (1 mg mL^–1^), vortexed, and filtered through Whatman paper for subsequent analyses.

### 2.5 Quantification of bioactive compounds

#### 2.5.1 Total phenolic content

Total phenolic content was determined following the method of Gurung (2020). A 20 μg of dried sample was placed in a 15 mL Falcon tube, followed by the addition of 1 mL distilled water, 2.5 mL of 20% sodium carbonate, and 500 μL of diluted Folin–Ciocalteu reagent (1:1 with water). The mixture was incubated in the dark for 40 minutes for color development. Absorbance was measured at 725 nm using a Multiskan GO microplate spectrophotometer (Thermo Fisher Scientific, Waltham, MA, USA) with a final volume of 200 μL per well. Gallic acid (0–100 ppm) was used for standard curve preparation, and results were expressed as mg gallic acid equivalents per gram of dry sample (mg GAE g^–1^).

#### 2.5.2 Total flavonoids content

Total flavonoid content was determined using the colorimetric method described by Yadav et al. (2024a). A 0.5 mL aliquot of dried sample solution (1 mg mL^–1^) was mixed with 1.5 mL methanol, 0.1 mL of 10% aluminum chloride, 0.1 mL of 1 M potassium acetate, and 2.8 mL distilled water. After vortexing, the mixture was incubated at room temperature for 30 minutes. Absorbance was measured at 415 nm using a microplate reader. Quercetin (0–600 μg/mL) was used to generate the standard curve, and results were expressed as mg quercetin equivalents per gram of dry sample (mg QE g^–1^).

#### 2.5.3 Total tannin content

Tannin content was estimated using the Folin-Denis method followed by Yadav et al. (2024a). A 0.1 mL aliquot of sample solution (1 mg mL^–1^) was mixed with 7.5 mL distilled water, 0.5 mL Folin-Denis reagent, and 1 mL of 35% sodium carbonate. The volume was adjusted to 10 mL with distilled water, vortexed, and incubated at room temperature for 30 minutes for color development. Absorbance was measured at 700 nm (final volume: 200 µL per microplate well). Tannic acid standards (0–100 μg/mL) were used for the calibration curve, and results were expressed as mg tannic acid equivalents per gram of dry sample (mg TAE g^–1^).

#### 2.5.4 Total carotenoids content, chlorophyll a, and chlorophyll b

Total carotenoid content was estimated following the method of Wellburn (1994). A 0.2 g dried sample was homogenized in 10 mL of absolute acetone, filtered through Whatman No. 1 filter paper, and centrifuged at 2500 rpm (875 × g) for 10 minutes (Eppendorf 5910 Ri, Germany). The supernatant was used to measure absorbance at 470 nm (carotenoids), 662 nm (Chl-a), and 645 nm (Chl-b) using a spectrophotometer. Chl-a, Chl-b, and total carotenoid contents (mg g^–1^ dry sample) were calculated using standard Equations 3–5.

(3) $$Chl-a=11.75\times A_{662}-2.35\times A_{645}$$

(4) $$Chl-b=18.61\times A_{645}-3.96\times A_{662}$$

(5) $$Total\;carotenoid\;content=\frac{1000\;\times \;A_{470}-2.27\;\times \;Chl-a-81.4\;\times \;Chl-b}{227}$$

### 2.6 Determination of antioxidant activities

#### 2.6.1 DPPH assay

The DPPH assay was conducted according to Brand-Williams et al. (1995). The reaction mixture included 10–150 µL of the sample, 3 mL of absolute ethanol, and 2 mL of 0.06 mM DPPH solution (in ethanol). A color changes from deep violet to light yellow indicated radical scavenging. After 30 minutes of incubation at room temperature, absorbance was measured at 517 nm using a spectrophotometer. Ethanol and DPPH solution served as the control. The percentage of DPPH scavenging activity was calculated according to Equation 6:

(6) $$DPPH\;scavenging\;activity\;\left(\%\right)=\left[\left(\frac{A_{0}-A_{1}}{A_{0}}\right)\times 100\right]$$

Where: *A*_0_ – absorbance of control, *A*_1_ = absorbance of sample.

#### 2.6.2 ABTS assay

The ABTS assay was performed following the method of Re et al. (1999). Equal volumes of 7.4 mM ABTS and 2.6 mM potassium persulfate were mixed and incubated in the dark for 12 hours to generate the ABTS radical. The resulting solution was diluted with methanol to an absorbance of 0.70 ± 0.02 at 734 nm. Then, 100 µL of the sample was mixed with 100 µL of the ABTS solution in a microplate well and incubated for 2 hours in the dark. Absorbance was recorded at 734 nm using a spectrophotometer. The percentage of ABTS+• was calculated according to Equation 7.

(7) $$ABTS\;scavenging\;activity\;\left(\%\right)=\left[\left(\frac{A_{0}-A_{1}}{A_{0}}\right)\times 100\right]$$

Where, *A*_0_ is the absorbance of the control; *A*_1_ is the absorbance of the sample.

#### 2.6.3 FRAP assay

FRAP activity was measured according to the method of Benzie and Strain (1996). The FRAP reagent was freshly prepared by mixing acetate buffer (300 mM, pH 3.6), 10 mM TPTZ solution in 40 mM HCl, and 20 mM FeCl_3_·6H_2_O in a 10:1:1 ratio, and incubated at 37 °C. A 100 µL sample was mixed with distilled water and FRAP reagent to a final volume of 200 µL per well in a microplate. After incubation, absorbance was recorded at 593 nm. A standard curve was prepared using FeSO_4_ solutions (0–1000 μg/mL), and results were expressed as µmol FeSO_4_ equivalents per gram of dry sample (µmol Fe^2+^ g^–1^ DW).

### 2.7 Ascorbic acid

Ascorbic acid content was estimated volumetrically following the method described by Yadav et al. (2024b). A standard solution was titrated with 2,6-dichlorophenol indophenol dye in 4% oxalic acid until a persistent pink endpoint appeared, and the dye volume was recorded as V_1_. For sample analysis, 0.5 g of dried powder was extracted with 10 mL of 4% oxalic acid, centrifuged, and 5 mL of the supernatant was titrated similarly. The dye volume used was noted as V_2_. The ascorbic acid content was calculated according to Equation 8:

(8) $$Ascorbic\;acid\;\left(mg\;100\;g^{-1}\right)=\left(\frac{0.5\;mg}{V_{1}ml}\right)\times \left(\frac{V_{2}}{15\;ml}\right)\times \left(\frac{100\;ml}{Wt.\;\;of\;sample}\right)\times 100$$

### 2.8 Statistical analysis

Experimental data were organized in Microsoft Excel and analyzed using one-way ANOVA in IBM SPSS Statistics v21.0 (IBM Corp., Armonk, NY, USA) to determine significant differences among treatment groups (p< 0.05). Where significant differences were found, Duncan’s multiple range test was performed as a *post hoc* analysis. Graphs and correlation matrix heatmap were generated using GraphPad Prism v9.5.1 (GraphPad Software, CA, USA).

## 3 Results

### 3.1 Effect of cow dung on biomass production

**Figure 1:**
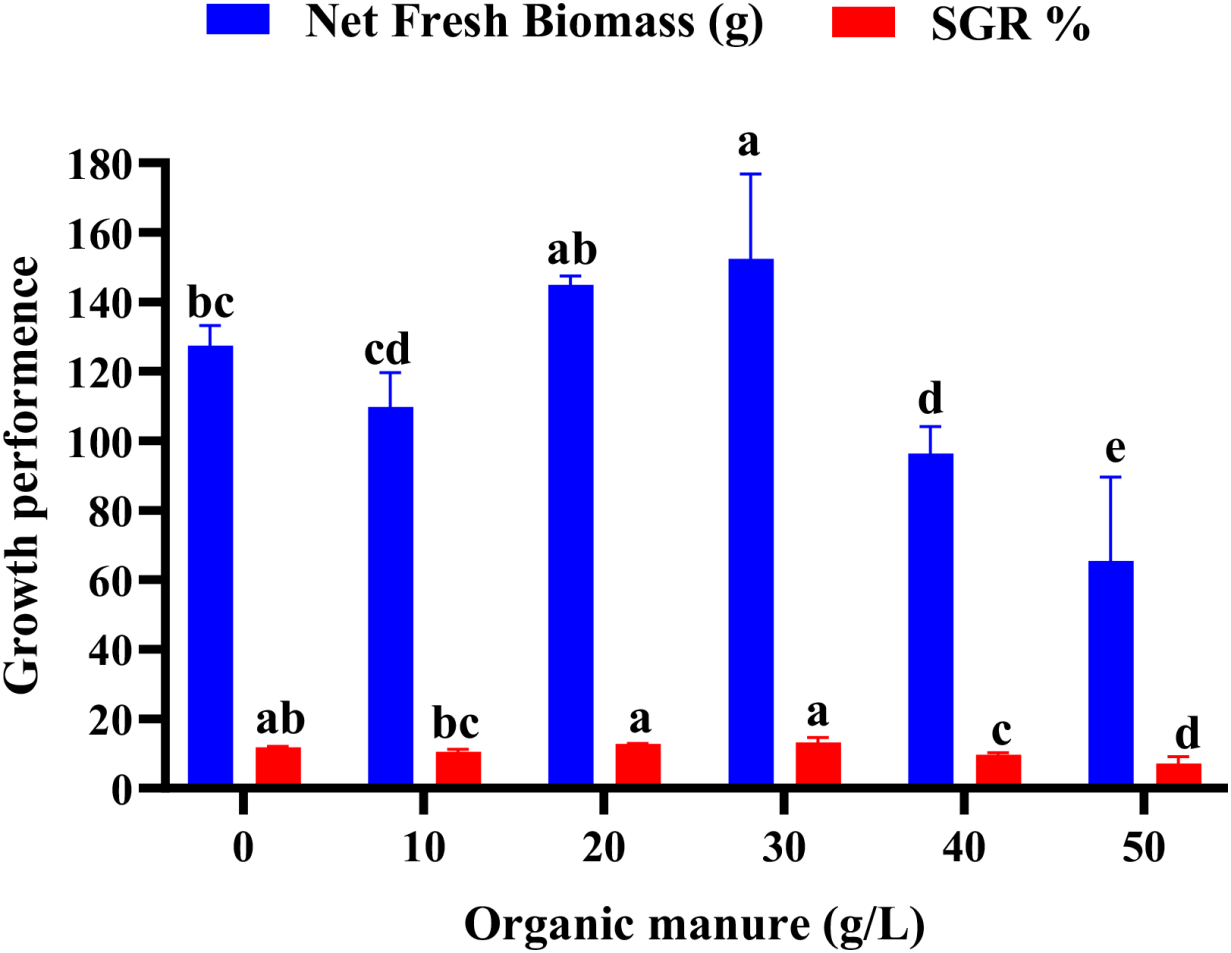
Net biomass production and specific growth rate of wolffia in response to different organic manure doses. Values are expressed as mean ± SD. Bars with different letters differed significantly (p*<* 0.05) among the experimental groups.

The effect of different doses of organic manure and inorganic fertilizer on wolffia biomass production and specific growth rate (SGR) is shown in **Figure 1**. Total biomass was highest at 30 and 20 g L^–1^ cow dung, reaching 152.38 ± 12.25 g and 144.88 ± 1.28 g, respectively, representing increases of approximately 19.6% and 13.7% over the control. These two treatments also produced the highest SGR values, indicating superior growth performance. In contrast, the lowest biomass (65.38 ± 12.11 g) was recorded at the highest cow dung concentration (50 g L^–1^), which was significantly lower than all other treatments. This suggests that excessive organic loading negatively affected wolffia biomass production.

### 3.2 Effect of cow dung on proximate composition

**Figure 2:**
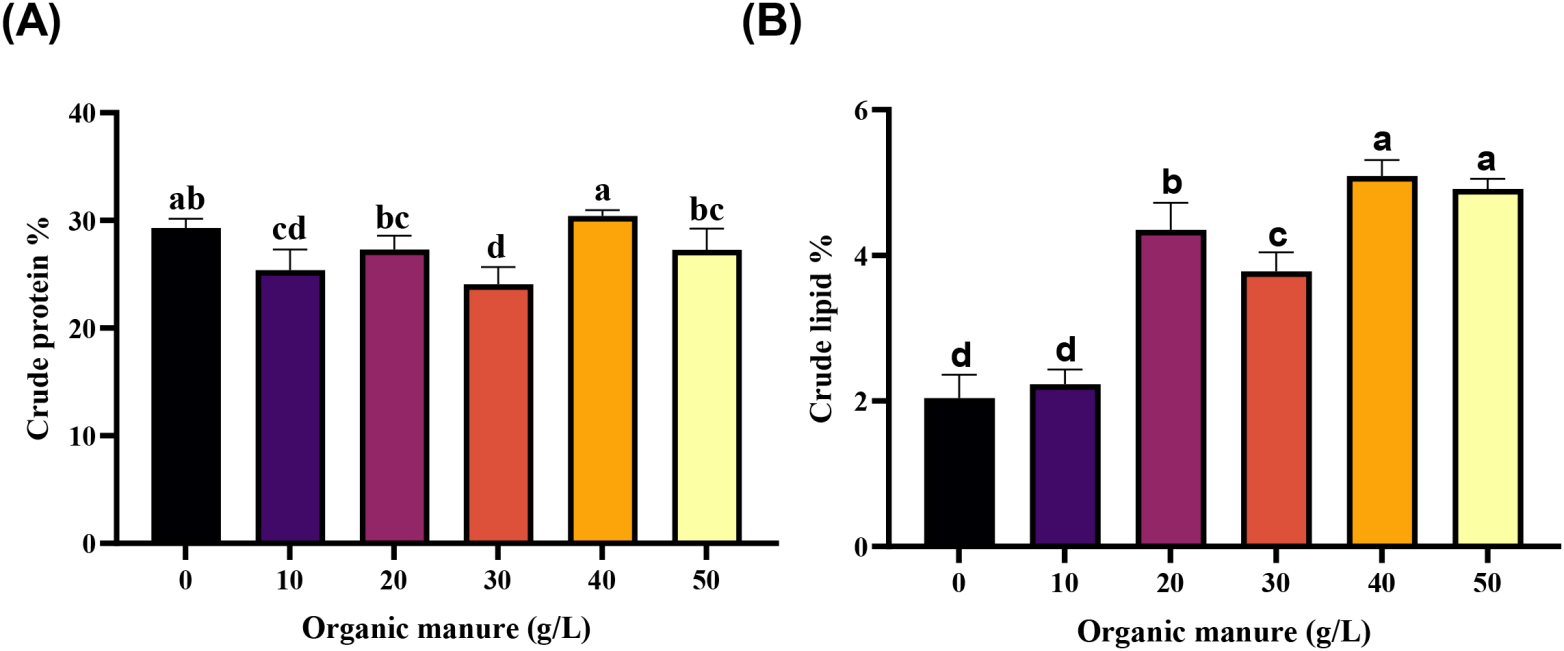
Proximate composition of wolffia subjected to different organic manure levels. **(A)** Crude protein, **(B)** Crude lipid. Data are represented as mean ± SD. Bars with different letters differed significantly (p*<* 0.05) among the experimental groups.

The proximate composition analysis of wolffia cultures showed significant variations in crude protein and lipid contents across treatments with inorganic fertilizer (control) and different doses of cow dung (**Figure 2**). The highest crude protein content was recorded in the 40 g L^–1^ cow dung treatment (30.40 ± 0.33%), which was significantly higher than all other organic manure treatments. The control group exhibited a comparable protein level (29.31 ± 0.48%), with no statistically significant difference from the 40 g L^–1^ treatment. Moderate protein levels were observed at 20 g L^–1^ (27.29 ± 0.74%) and 50 g L^–1^ (27.28 ± 1.14%), while the lowest values occurred at 10 g L^–1^ (25.39 ± 1.10%) and 30 g L^–1^ (24.08 ± 0.91%).

In terms of crude lipid content, the highest values were found in the 40 g L^–1^ (5.09 ± 0.12%) and 50 g L^–1^ (4.91 ± 0.08%) cow dung treatments. These values align closely with reported lipid levels in wolffia species (~5.3% dry weight). The lowest lipid contents were observed in the control (2.04 ± 0.19%) and 10 g L^–1^ (2.23 ± 0.12%) treatments.

### 3.3 Effect of cow dung on bioactive compounds

#### 3.3.1 Total phenolic content

**Figure 3:**
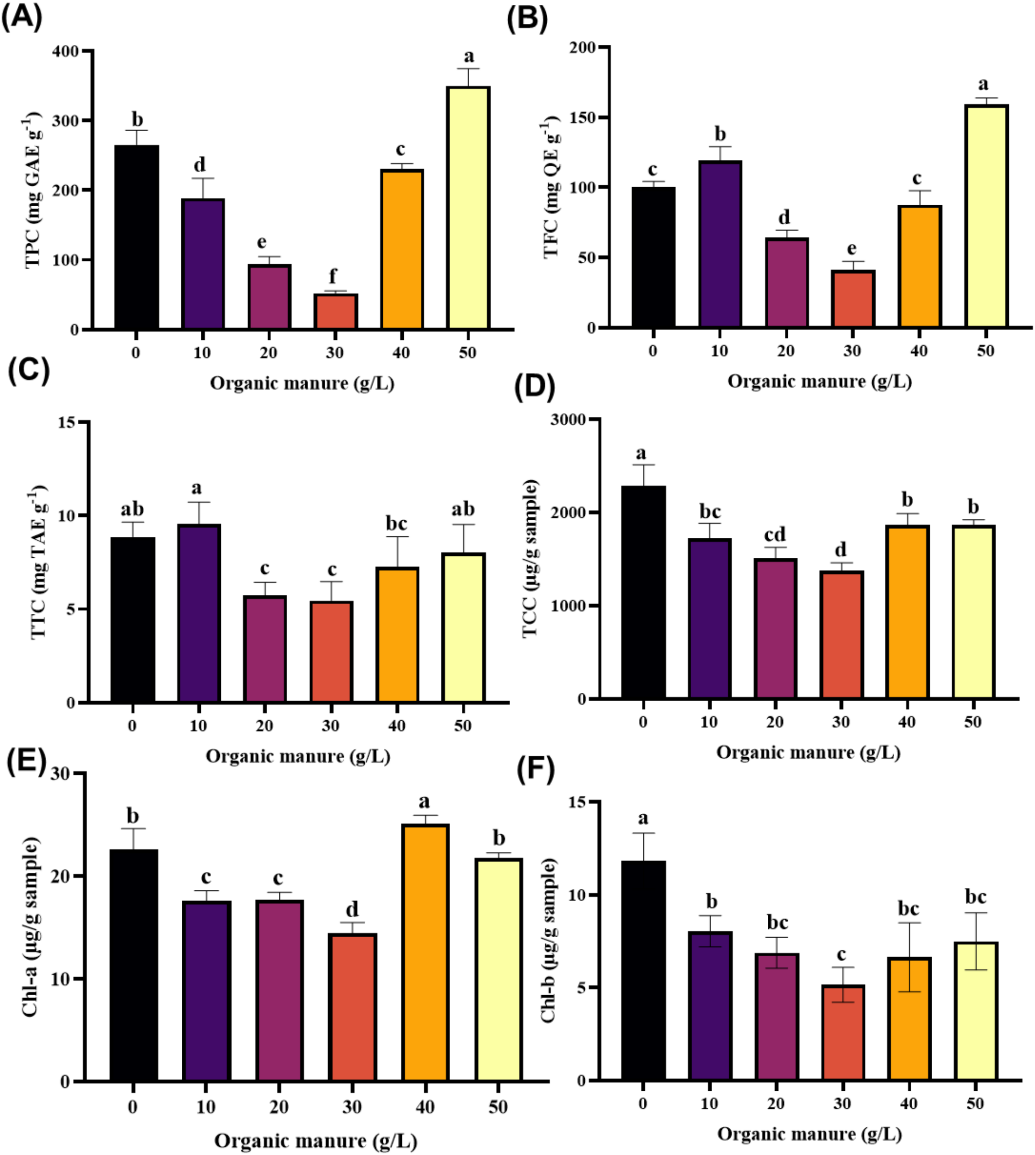
Effect of organic manure on bioactive compounds of wolffia. **(A)** Total phenolic content, **(B)** Total flavonoid content, **(C)** Total tannin content, **(D)** Total carotenoid content, **(E)** Chlorophyll-a, **(F)** Chlorophyll-b. Data are represented as mean ± SD. Bars with different letters differed significantly (p*<* 0.05) among the experimental groups.

The total phenolic content (TPC) of wolffia under various cow dung treatments is shown in **Figure 3A**. A significant variation (p< 0.05) was observed across treatments. The highest TPC was recorded at 50 g L^–1^ cow dung, yielding 350.04 ± 14.27 mg GAE g^–1^, followed by the control (inorganic fertilizer) with 265.61 ± 11.88 mg GAE g^–1^. In contrast, the lowest TPC was observed at 30 g L^–1^ cow dung (52.21 ± 2.02 mg GAE g^–1^). Compared to the control, the 50 g L^–1^ treatment resulted in a 31.79% increase in TPC and was significantly higher by 52.05%, 85.42%, 273.35%, and 570.27% relative to the 40, 10, 20, and 30 g L^–1^ cow dung treatments, respectively.

#### 3.3.2 Total flavonoid content

The total flavonoid content (TFC) of wolffia cultured under different doses of cow dung and inorganic fertilizer is shown in **Figure 3B**. A significant difference (p< 0.05) was observed among treatments. The highest TFC was recorded at the 50 g L^–1^ cow dung dose, with a value of 159.35 ± 2.59 mg QE g^–1^, followed by the 10 g L^–1^ treatment (119.32 ± 5.63 mg QE g^–1^). The lowest TFC (41.09 ± 3.60 mg QE g^–1^) occurred at the 30 g L^–1^ cow dung dose.

#### 3.3.3 Total tannin content

The total tannin content (TTC) of wolffia cultured under varying doses of cow dung and inorganic fertilizer is shown in **Figure 3C**. Tannin content did not differ significantly among the control (8.87 ± 0.46 mg TAE g^–1^), 10 g L^–1^ (9.55 ± 0.68 mg TAE g^–1^), and 50 g L^–1^ (8.03 ± 0.87 mg TAE g^–1^) cow dung treatments. In contrast, significantly (p > 0.05) lower TTC values were observed at 20 g L^–1^ (5.73 ± 0.41 mg TAE g^–1^) and 30 g L^–1^ (5.46 ± 0.59 mg TAE g^–1^). The 40 g L^–1^ treatment resulted in intermediate TTC (7.24 ± 0.95 mg TAE g^–1^), which did not differ significantly from the 50 g L^–1^ treatment but was lower than the control and 10 g L^–1^ treatments. Overall, moderate cow dung doses (20–30 g L^–1^) led to a decline in tannin accumulation in wolffia.

#### 3.3.4 Total carotenoids content, chlorophyll-a, and chlorophyll-b

The effects of inorganic fertilizer and different doses of cow dung on TCC, Chl-a, and Chl-b in wolffia are shown in **Figures 3D–F**. TCC varied significantly among treatments. The highest TCC was observed in the control group (2286.90 ± 130.93 µg g^–1^), followed by the 40 g L^–1^ (1868.04 ± 31.75 µg g^–1^) and 50 g L^–1^ (1864.45 ± 71.31 µg g^–1^) cow dung treatments, which did not differ significantly from each other but were significantly lower than the control (**Figure 3D**). The 10 g L^–1^ treatment also produced relatively high carotenoid levels (1722.46 ± 93.23 µg g^–1^), significantly higher than the 20 g L^–1^ (1507.36 ± 69.12 µg g^–1^) and 30 g L^–1^ (1377.83 ± 47.91 µg g^–1^) treatments, which recorded the lowest TCC values.

The Chl-a content was highest at the 40 g L^–1^ dose (25.14 ± 0.47 mg g^–1^), which was significantly higher than all other treatments (**Figure 3E**). Relatively high Chl-a levels were also recorded in the control (22.61 ± 1.17 mg g^–1^) and 50 g L^–1^ (21.77 ± 0.30 mg g^–1^) treatments. Lower Chl-a contents were observed in the 10 g L^–1^ (17.63 ± 0.57 mg g^–1^) and 20 g L^–1^ (17.73 ± 0.42 mg g^–1^) treatments, while the 30 g L^–1^ treatment had the lowest Chl-a concentration (14.49 ± 0.59 mg g^–1^), significantly lower than all other treatments.

Chl-b content also showed significant variation among treatments (**Figure 3F**). The highest Chl-b level occurred in the control group (11.82 ± 0.87 mg g^–1^), followed by the 10 g L^–1^ cow dung treatment (8.04 ± 0.49 mg g^–1^). Both were significantly higher than all other treatments. Moderate Chl-b values were recorded at 20 g L^–1^ (6.88 ± 0.48 mg g^–1^) and 50 g L^–1^ (7.49 ± 0.89 mg g^–1^), while the lowest Chl-b concentration was observed at 30 g L^–1^ (5.15 ± 0.55 mg g^–1^).

### 3.4 Effects of organic manure on antioxidant activities of wolffia

#### 3.4.1 DPPH assay

**Figure 4:**
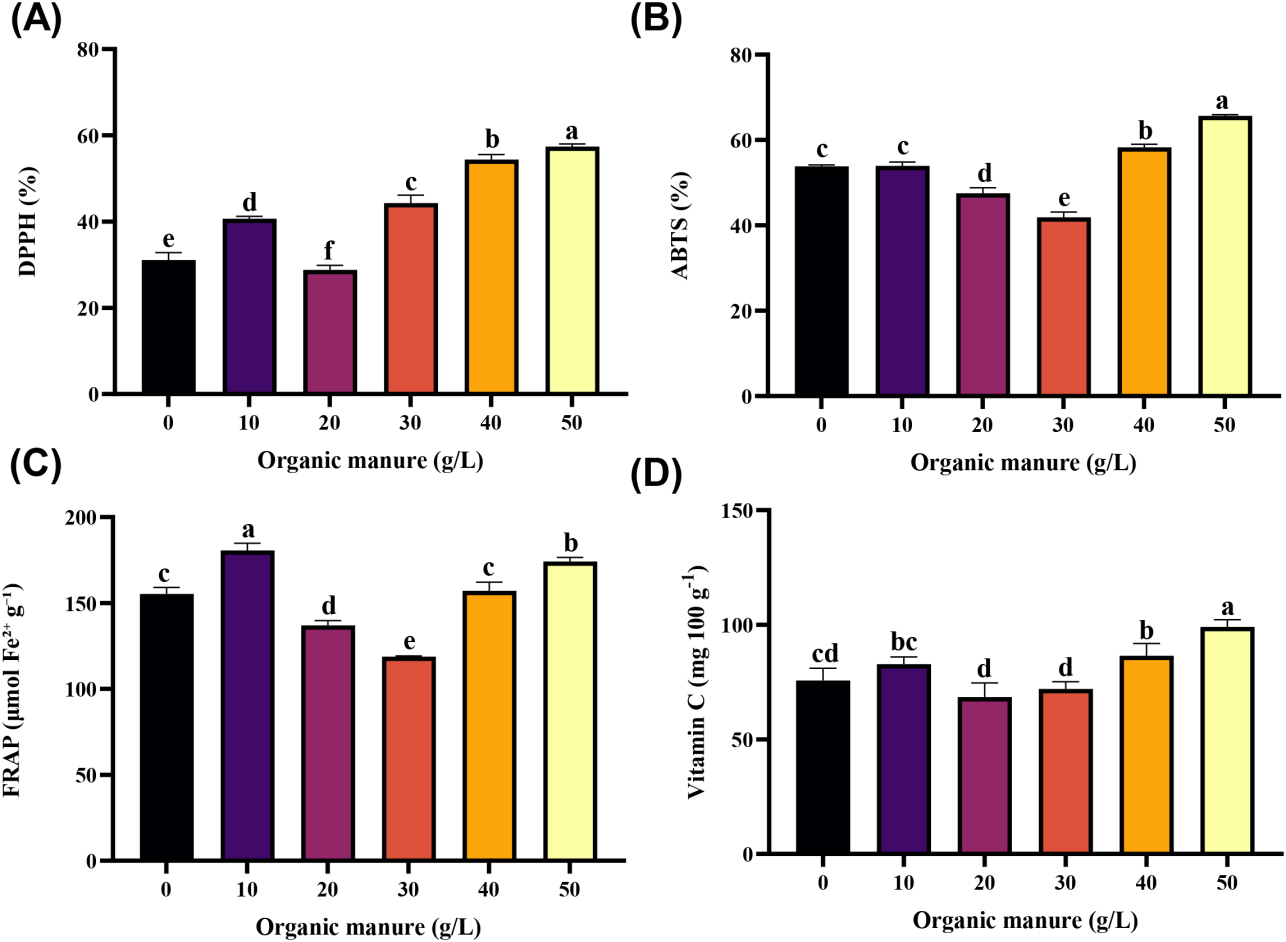
Effect of organic manure on antioxidant activities of wolffia. **(A)** DPPH scavenging activity, **(B)** ABTS scavenging activity, **(C)** FRAP activity, **(D)** Vitamin **(C)** Data are represented as mean ± SD. Bars with different letters differed significantly (p*<* 0.05) among the experimental groups.

Antioxidant activity differed significantly among fertilizer treatments. As shown in **Figure 4A**, the highest DPPH radical scavenging activity was recorded at the 50 g L^–1^ cow dung dose (57.42 ± 0.33%), followed by the 40 g L^–1^ treatment (54.39 ± 0.68%). The lowest DPPH activity was observed at 20 g L^–1^ (28.82 ± 0.59%). These results indicate that higher concentrations of organic manure enhanced the antioxidant capacity of wolffia.

#### 3.4.2 ABTS assay

The ABTS radical scavenging activity of wolffia under different fertilizer treatments is presented in **Figure 4B**. The highest ABTS activity was recorded in the 50 g L^–1^ cow dung treatment (65.65 ± 0.18%), followed by the 40 g L^–1^ dose (58.27 ± 0.42%). The lowest ABTS activity occurred at the 30 g L^–1^ treatment, with a value of 41.90 ± 0.71%. These results indicate that higher levels of cow dung application substantially improved ABTS antioxidant capacity in wolffia.

#### 3.4.3 FRAP assay

The FRAP antioxidant activity of wolffia varied significantly across treatments with different doses of inorganic and organic fertilizers. The highest FRAP value was observed at the 10 g L^–1^ cow dung treatment (180.64 ± 2.40 μmol Fe g^–1^ dry mass), followed by the 50 g L^–1^ dose (174.18 ± 1.37 μmol Fe g^–1^ dry mass). These values represent increases of approximately 16.30% and 12.14%, respectively, compared to the control (155.32 ± 2.19 μmol Fe g^–1^ dry mass), as shown in **Figure 4C**. These results indicate that lower to higher doses of cow dung improved FRAP antioxidant capacity relative to inorganic fertilizer.

### 3.5 Ascorbic acid

The Vitamin C (Vit C) content of wolffia cultured under different doses of inorganic and organic fertilizers is shown in **Figure 4D**. The highest Vit C content was observed at the 50 g L^–1^ cow dung dose, reaching 99.05 ± 1.80 mg 100 g^–1^, representing a 30.97% increase compared to the control (75.63 ± 3.12 mg 100 g^–1^). This was followed by the 40 g L^–1^ treatment (86.44 ± 3.12 mg 100 g^–1^) and the 10 g L^–1^ dose (82.84 ± 1.80 mg 100 g^–1^). These results demonstrate that increasing levels of organic fertilizer application enhanced Vit C accumulation in wolffia.

### 3.6 Correlation analysis between bioactive compound and antioxidant activities

**Figure 5:**
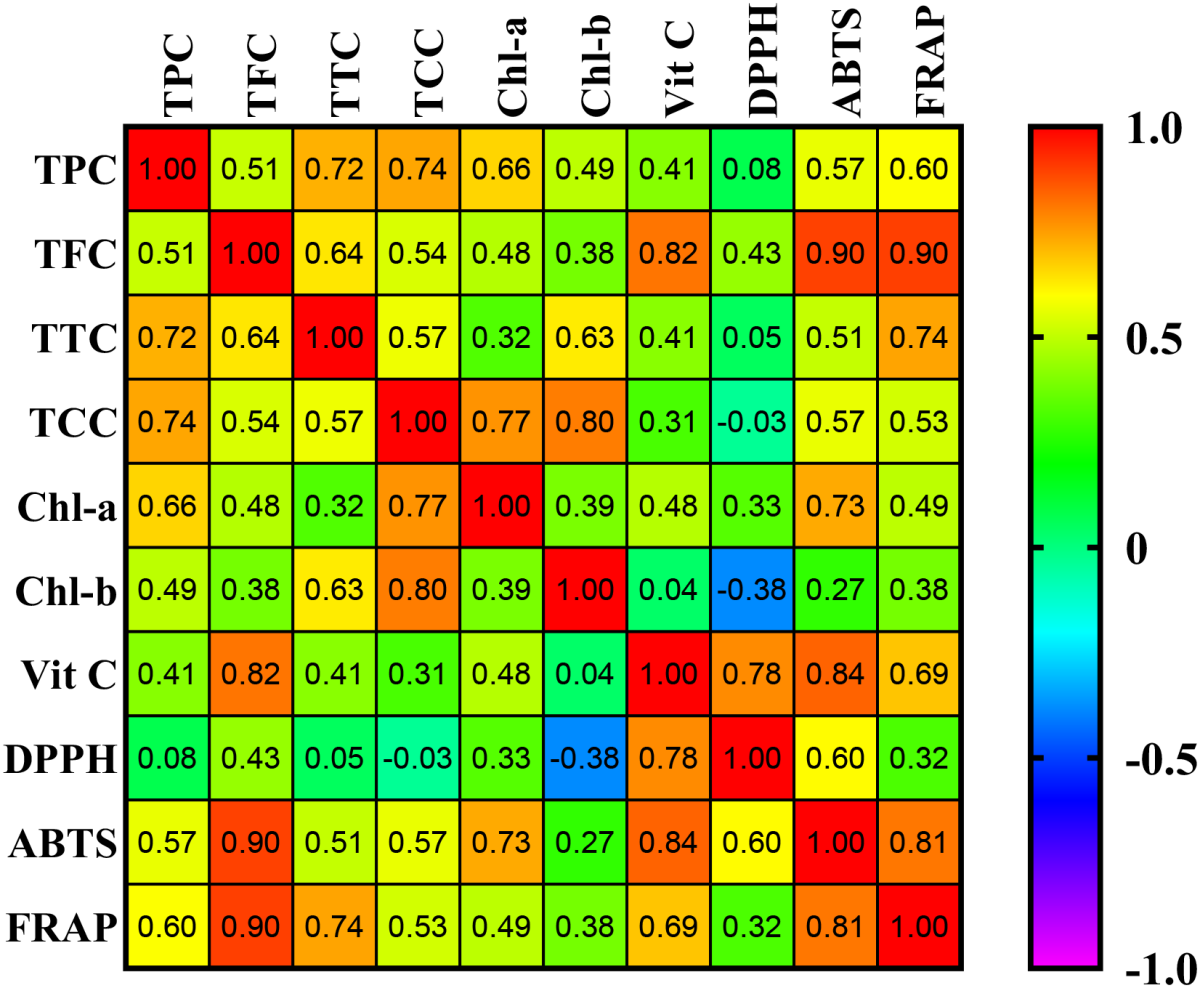
Correlation analysis of the bioactive compounds and antioxidant activities of wolffia grown under different levels of cow dung. It ranges from –1 to 1, whereby–1 means a perfect negative linear relationship between variables, 1 indicates a perfect positive linear relationship between variables and 0 indicates that there is no relationship between studied variables. Significant level (p< 0.05).

The Pearson correlation analysis between bioactive compounds and antioxidant activities revealed several significant associations (**Figure 5**). TPC showed positive correlations with DPPH (r = 0.08), ABTS (r = 0.57), and FRAP (r = 0.60). TFC exhibited strong positive correlations with ABTS (r = 0.90), FRAP (r = 0.90), and Vit C (r = 0.82). TTC was positively correlated with DPPH (r = 0.05), ABTS (r = 0.51), FRAP (r = 0.74) and Vit C (r = 0.41). Additionally, TCC showed positive correlations with ABTS (r = 0.57) and FRAP (r = 0.53), while displaying a slight negative correlation with DPPH (r = –0.03). Overall, the correlation pattern indicates that higher concentrations of phenolic and flavonoid compounds are generally associated with stronger antioxidant activity.

## 4 Discussion

### 4.1 Effect of cow dung on biomass production

The enhanced biomass production observed at 20 and 30 g L^–1^ cow dung indicates that moderate organic manure levels effectively support early wolffia growth, likely through increased microbial activity that enhances nutrient mineralization and phytohormone release (Mofunanya et al., 2015). In contrast, the marked reduction in biomass at the highest cow dung concentration (50 g L^–1^) can be attributed to excessive manure loading, which elevates ammonia (NH_3_) levels and exerts toxic effects on aquatic plants (Sońta et al., 2023). Similar trends have been reported by Stadtlander et al. (2022), who observed higher fresh biomass yields of duckweed (*Spirodela polyrhiza* and *Lemna punctata*) at lower bovine slurry concentrations. In a subsequent study, the same authors further demonstrated that while solubilized chicken manure enhanced the growth of *Lemna minor* at optimal doses, higher concentrations led to plant die-off due to ammonium-nitrogen (NH_4_–N) toxicity (Stadtlander et al., 2023).

### 4.2 Effect of cow dung on proximate composition

The higher crude protein content observed at the 40 g L^–1^ cow dung dose indicates that this level provides an optimal nutrient supply to support protein synthesis in wolffia. This aligns with previous reports showing that wolffia species typically contain 20–30% crude protein (Appenroth et al., 2018; Chantiratikul et al., 2010; Baidya and Patel, 2017). The enhancement in protein content at optimal organic manure levels can be attributed to the steady release of nitrogen during organic matter decomposition, which promotes amino acid and protein synthesis (Han et al., 2022).

Similarly, the elevated crude lipid content observed at 40 and 50 g L^–1^ cow dung reflects improved availability of key nutrients, particularly N, P, and K, which are essential for lipid biosynthesis and metabolic activity (Gong et al., 2020). The lipid levels recorded in this study are consistent with earlier findings in wolffia (~5.3% dry weight; Said et al., 2022a) and are substantially higher than those reported for other duckweed species such as *Spirodela polyrhiza* (~1.74%; Said et al., 2022b). Overall, these results demonstrate that appropriately optimized organic manure application enhances both protein and lipid accumulation in wolffia, whereas insufficient nutrient inputs limit these biochemical processes.

### 4.3 Effect of cow dung on bioactive compounds

Bioactive compounds, particularly phenolic compounds in plants, have received increasing attention due to their diverse health benefits and biological activities (Yadav et al., 2024a). In the present study, TPC increased markedly at the higher cow dung dose (50 g L^–1^), indicating that elevated organic manure levels can stimulate phenolic biosynthesis in wolffia. This response is likely associated with organic manures enhancing shikimic acid production, thereby promoting the biosynthesis of phenolic compounds and flavonoids (Darakeh et al., 2021; Sousa et al., 2008). Similar stimulatory effects of organic fertilization on phenolic enrichment have been reported in other plant species. For instance, Naguib et al. (2012) documented a 51.04% increase in total phenolics in broccoli grown under combined organic and inorganic fertilization, while Ibrahim et al. (2013) reported elevated secondary metabolite content and antioxidant activity in *Labisia pumila* under higher organic nitrogen inputs.

Flavonoids, which are key antioxidant secondary metabolites, contribute substantially to oxidative stress mitigation due to their hydroxyl and carboxyl functional groups (Yadav et al., 2024a). In the present study, the elevated TFC observed at 50 g L^–1^ cow dung further supports the role of organic manure in stimulating flavonoid biosynthesis. The supply of readily available nitrogen and organic carbon from cattle dung likely enhanced metabolic flux toward phenolic and flavonoid synthesis (Villamarin-Raad et al., 2023), a response commonly associated with activation of the shikimate–phenylpropanoid pathway, particularly through increased phenylalanine ammonia-lyase (PAL) activity (Bayat et al., 2021). Comparable increases in flavonoid accumulation following organic fertilization have been reported in *Cichorium intybus* (Gholami et al., 2018) and across different plant morphotypes treated with cattle dung (Oloyede and Oyelade, 2022). Together, these observations confirm that cow dung application enhances metabolic activity associated with secondary metabolite formation, underscoring the nutraceutical potential of wolffia cultivated under higher cow dung doses.

Total tannin content showed a non-linear response to cow dung application, with comparable levels in the control, 10, and 50 g L^–1^ treatments, and reduced accumulation at intermediate doses. The reduced tannin content at intermediate doses likely reflects enhanced growth-driven N allocation to biomass; under the C/N balance theory, N sufficiency favors growth over the synthesis of carbon-based, nitrogen-free tannins (Libutti et al., 2023). Partial recovery of tannin levels at higher doses indicates that wolffia may re-establish metabolic balance once sufficient nutrients become available. These findings are consistent with Oluwole et al. (2022), who reported increased tannin accumulation in *Launaea taraxacifolia* following cow dung amendment, underscoring the importance of dosage in regulating tannin responses.

Photosynthetic pigment responses varied with treatments. The significantly higher TCC and Chl-b levels in the control indicate that inorganic fertilizer promoted more efficient pigment accumulation, likely due to the rapid availability of nutrients. Because chlorophyll synthesis is closely linked to nitrogen availability, the readily available nitrogen supplied by inorganic fertilizer enhanced pigment accumulation and photosynthetic capacity compared with cow dung treatments (Ndirmbula et al., 2022). Lower pigment levels at 20–30 g L^–1^ cow dung reflect a growth–dilution effect, where rapid biomass accumulation and preferential nitrogen allocation toward growth reduce pigment concentration despite enhanced productivity (Poorter et al., 2022). However, the highest Chl-a content at 40 g L^–1^ cow dung indicates that moderate to higher organic inputs can enhance photosynthetic pigment synthesis when nutrient supply is adequate. Organic manure provides a gradual release of nitrogen, a key element for chlorophyll formation, although its release dynamics differ from inorganic sources. Similar enhancements in chlorophyll content under high organic and inorganic fertilization have been reported in *Foeniculum vulgare* (Nada et al., 2022). Overall, these results demonstrate that both inorganic fertilizers and appropriately optimized organic manure doses can improve pigment composition in wolffia, with responses varying according to pigment type and nutrient source.

### 4.4 Effects of organic manure on antioxidant activities of wolffia

The enhanced DPPH and ABTS radical scavenging activities observed at higher cow dung doses reflect the physiological responses of wolffia under organic nutrient regimes. Organic manures such as cow dung mineralize slowly and are generally less nutrient-dense than inorganic fertilizers, which can result in transient nutrient limitation during critical growth stages (Seufert et al., 2012). Such conditions may induce oxidative stress, leading to increased production of reactive oxygen species (ROS) and activation of antioxidant defense systems, including enzymes such as superoxide dismutase (Chang et al., 2008). Excess ROS disrupt cellular homeostasis by inhibiting enzymatic activity and damaging membranes (Luis, 2015), thereby stimulating defense mechanisms that promote the synthesis of secondary metabolites, particularly phenolic compounds and flavonoids (Vallverdu-Queralt et al., 2012; Sharma et al., 2012). These metabolites possess strong antioxidant properties and effectively scavenge free radicals, contributing to cellular protection against oxidative stress (Rostaei et al., 2024). Consistent with this mechanism, treatments exhibiting higher total phenolic and flavonoid contents also showed greater ABTS activity, in agreement with observations reported by Liwanda et al. (2023) in cow manure–fertilized purslane.

The enhanced FRAP activity at 10 g L^–1^ cow dung–treated wolffia further supports the role of organic manure in strengthening antioxidant capacity. The slow and sustained nutrient release characteristic of organic fertilizers promotes prolonged activation of defense responses and gradual accumulation of reducing compounds involved in oxidative stress mitigation (Rostaei et al., 2018). Similar increases in FRAP activity following cow manure application have been reported by Liwanda et al. (2023), reinforcing the role of cow dung in enhancing antioxidant potential through sustained physiological and metabolic adjustments.

### 4.5 Ascorbic acid

Vit C is a water-soluble antioxidant that plays a crucial role in protecting human health. In the present study, higher Vit C levels were observed in wolffia under organic manure treatment, particularly at 50 g L^–1^ cow dung, likely due to improved nutrient availability and uptake that support ascorbic acid biosynthesis. Organic manure decomposes slowly, ensuring a gradual release of macro- and micronutrients (e.g., N, P, and iron) that serve as cofactors or substrates in metabolic pathways involved in ascorbic acid production (Wu et al., 2024). These findings are consistent with earlier reports demonstrating enhanced Vit C accumulation under organic fertilization. For instance, Wang et al. (2010) reported increased Vit C content in Chinese cabbage grown with a cow manure vermicompost–soil mixture, while Hassan et al. (2012) observed higher Vit C levels in *Cosmos caudatus* under organic fertilizer application compared with inorganic inputs. Similarly, Toor et al. (2006) showed that organic fertilizers, including chicken manure and grass–clover, increased Vit C content in tomatoes while reducing nitrate accumulation, and Asami et al. (2003) documented enhanced Vit C levels in marionberry, strawberry, and corn under organic fertilization. Collectively, these studies support the present findings, indicating that organic fertilizers particularly cow dung promote Vit C synthesis by improving nutrient balance and supporting secondary metabolic pathways.

### 4.6 Correlation analysis between bioactive compound and antioxidant activities

The positive correlations observed between bioactive compounds particularly phenolics and flavonoids and antioxidant activities support the role of these metabolites as major contributors to the antioxidant potential of wolffia. The strong correlations of TFC with ABTS, FRAP, and Vit C suggest that flavonoids act as potent electron donors and radical scavengers, thereby enhancing both radical neutralization and reducing power.

The moderate to strong correlations of TPC and TTC with antioxidant assays further highlight the importance of polyphenolic compounds in mitigating oxidative stress. These findings align with Carvalho et al. (2015), who reported similar positive correlations between phenolics and antioxidant activities in Capsicum species, measured through ABTS and DPPH assays. Additionally, the overall positive association between polyphenol concentrations and antioxidant potential supports the observations of Yadav et al. (2024b), reinforcing the notion that phenolics and flavonoids serve as key determinants of antioxidant activity in plant systems. The slight negative correlation between TCC and DPPH may suggest differing mechanisms or efficiencies of carotenoid-mediated antioxidant activity in this specific assay; however, the positive correlations with ABTS and FRAP indicate that carotenoids still contribute meaningfully to overall antioxidant capacity.

## 5 Conclusion

This study demonstrates that organic manure, particularly cow dung at 50 g L^–1^, markedly enhances the biochemical and functional attributes of wolffia. While inorganic fertilizer supported higher pigment accumulation in some cases, the 50 g L^–1^ cow dung treatment consistently promoted superior levels of bioactive compounds including total phenolics, flavonoids, carotenoids, and vitamin C and resulted in significantly higher antioxidant activities (DPPH, ABTS, and FRAP). These improvements reflect the plant’s enhanced metabolic response under organic nutrient regimes, likely driven by sustained nutrient release and the activation of defense pathways associated with secondary metabolite synthesis.

Overall, the findings highlight that fresh cow dung concentration should be strategically optimized according to production objectives, with 20–30 g L^–1^ recommended for maximizing biomass yield and ~50 g L^–1^ for enhancing bioactive compounds and antioxidant potential in wolffia, thereby increasing its value for functional food and aquaculture applications. Future research should focus on detailed fatty acid profiling, targeted identification of individual phenolic compounds, and elucidation of underlying metabolic pathways to better understand the health-promoting properties and industrial potential of organically cultivated wolffia.

## Author's note

*Wolffia globosa* was collected from the pilot-scale culture pond at the College of Fisheries, Central Agricultural University (Imphal), Tripura, India. This species is not listed as endangered or threatened according to the International Union for Conservation of Nature (IUCN) red list of threatened species and is not covered by the convention on the trade in endangered species of wild fauna and flora. Additionally, all relevant guidelines for plant handling were followed.

## Data Availability

The original contributions presented in the study are included in the article/supplementary material. Further inquiries can be directed to the corresponding authors.
